# Molecular dynamics study on micelle-small molecule interactions: developing a strategy for an extensive comparison

**DOI:** 10.1007/s10822-023-00541-1

**Published:** 2023-12-16

**Authors:** Aleksei Kabedev, Christel A. S. Bergström, Per Larsson

**Affiliations:** 1 Department of Pharmacy, Uppsala University, Uppsala, Sweden; 2 Swedish Drug Delivery Center, Uppsala University, Uppsala, Sweden

**Keywords:** Molecular dynamics simulations, Solubilization, Extensive screening, Umbrella sampling, Intestinal fluid

## Abstract

Theoretical predictions of the solubilizing capacity of micelles and vesicles present in intestinal fluid are important for the development of new delivery techniques and bioavailability improvement. A balance between accuracy and computational cost is a key factor for an extensive study of numerous compounds in diverse environments. In this study, we aimed to determine an optimal molecular dynamics (MD) protocol to evaluate small-molecule interactions with micelles composed of bile salts and phospholipids. MD simulations were used to produce free energy profiles for three drug molecules (danazol, probucol, and prednisolone) and one surfactant molecule (sodium caprate) as a function of the distance from the colloid center of mass. To address the challenges associated with such tasks, we compared different simulation setups, including freely assembled colloids versus pre-organized spherical micelles, full free energy profiles versus only a few points of interest, and a coarse-grained model versus an all-atom model. Our findings demonstrate that combining these techniques is advantageous for achieving optimal performance and accuracy when evaluating the solubilization capacity of micelles.

**Graphical abstract:**

All-atom (AA) and coarse-grained (CG) umbrella sampling (US) simulations and point-wise free energy (FE) calculations were compared to their efficiency to computationally analyze the solubilization of active pharmaceutical ingredients in intestinal fluid colloids.

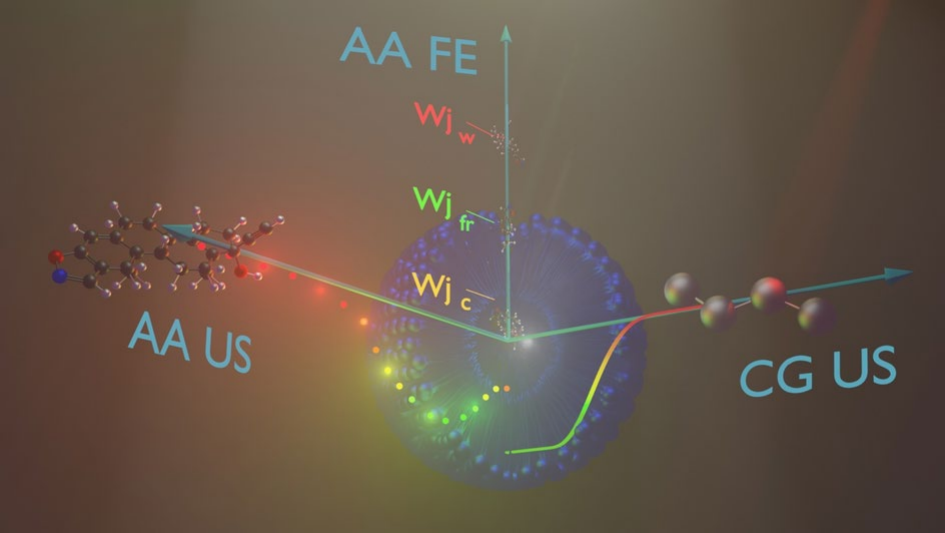

**Supplementary Information:**

The online version contains supplementary material available at 10.1007/s10822-023-00541-1.

## Introduction

Colloidal structures play an important role in the digestion and absorption of drug molecules in the gastrointestinal tract [1–3]. For poorly soluble drugs, solubilization by these structures is one of the key mechanisms that prevent aggregation, nucleation, and recrystallization [4, 5]. Solubilization normally occurs in the intestinal lumen, where a substance dissolves in fluid before passing through the cell membrane on its way to the circulatory system. In 1998, Dressmann et al*.* published an article on the composition of biorelevant fluids to mimic intestinal fluids in in vitro experiments [6]. Bile salts (BS), such as sodium taurocholate, when mixed with lecithin, water, and a few other minor components, form a dissolution medium that shows good compatibility with experimental data regarding composition, volume, and hydrodynamics. Several other versions of such simulated intestinal fluids are based on mixing BS and phospholipids (PL) at different ratios [7–9]. Aspiration studies involving healthy human volunteers indicated high variability in the concentration levels of BS and PL, but their ratio remained approximately four-to-one in the fasted state [10]. Therefore, micelles with a 4:1 BS-PL ratio can serve as a good model for an average colloid with which small molecules interact in the small intestine. Studies on the interactions of such micelles with drug molecules, excipients, and co-solvents would enable a better understanding of the solubilization and permeation processes, and enable the determination of drug solubility and permeability. Advanced formulations such as amorphous solid dispersions and lipid-based formulations also interact with APIs in complex ways. Thus, there is a need to study the affinity of drugs for carriers, their stability, mobility, drug release, and other related factors, to better understand and improve the delivery of drug molecules. However, experimental studies for such purposes can be time-consuming, and it is often not possible to pinpoint the nature of the specific molecular interactions that are important for solubilization.

Based on computational simulations, theoretical predictions could fill this gap and facilitate early screening of drug-intestinal fluid colloid interactions. Calculating the free energy (FE) changes associated with drug solubilization in micelles and membranes is a common technique for computationally studying such interactions [11]. Molecular dynamics (MD) allows one to estimate FE and simultaneously study the underlying mechanisms of drug solubilization. Measuring the solvation FE for one drug molecule in a variety of solvents [12] and media or for various drug molecules in the same environment [13] can be used to predict relative solubility. Umbrella sampling (US) and other enhanced sampling methods can be used to calculate the FE profile of a transition from one medium to another [14–16]. Thus, all-atom (AA) MD coupled with US is a powerful tool for studying the solubilization of small molecules in micelles. However, high-throughput screening based on AA FE calculations is computationally expensive. Some groups have proposed combining short MD simulations and machine learning (ML) to predict solvation FE [17–19]. For example, Riniker used simulations of molecules in vacuum and water as a basis for molecular dynamics fingerprints, which served as input for ML [18]. Although this approach is efficient, it cannot easily predict solvation in multicomponent media. Nevertheless, free energy profiles are a valuable source of information on their own and as input data for ML models. Therefore, it would be beneficial to introduce a relatively inexpensive MD simulation protocol capable of reflecting the interplay of drugs with complex gastrointestinal fluids, excipients, co-solvents, and surfactants. It is essential for the MD protocol to produce free energy profiles that are robust, sufficiently accurate, and cost-efficient in terms of computational resources.

Intuitively, micelles can be represented as self-assembled colloids equilibrated with respect to their aggregation number in a previous simulation. In several studies, this approach has been used as a basis for examining micelle-small molecule interactions [20–23]. The molecule is typically artificially pulled out from either the colloid center of mass or from a position corresponding to the minimum free energy (for instance, the surface of the colloid) and outwards to the bulk. In spite of this method being straightforward and enables umbrella sampling from unbiased colloidal structures, it has a drawback in that drugs and surfactants may interact with the same colloid in multiple ways, leading to variability in their shape and composition. Accounting for these interactions without multiple simulations is a challenging task. Additionally, if the molecule being pulled is either large or reasonably hydrophobic, micelle disintegration can occur. Finally, if the colloid is not spherical, another sampling problem might also be present: the free energy profile through the semi-minor and semi-major axes (or any other direction) will most likely look different. Such issues can lead to difficulties in accurately comparing the different solubilization patterns.

A reduction in the number of degrees of freedom of the system can be applied for a fast and unified, but arguably less realistic comparison of the free energy profiles. For example, position restraints can be set to limit the displacement of the micelle components while the small molecule is being pulled. In addition, an ideally spherical micelle loaded with one small molecule can first be built, where the ends of the micelle components are restrained with a flat-bottom potential [20]. In this manner, the atoms of the micelles can still move slightly, giving way to the molecule being pulled, whereas the structure of the micelle is predefined. In principle, the free energy profile for a drug in such a colloid is isotropic with respect to the micelle shape. The resulting free energy profiles can then provide quantitatively comparable information on the affinity of small molecules to all layers of colloidal structures.

However, the problem of high computational cost remains even in idealized isotropic systems. Given the typical size of micelles relevant to luminal intestinal fluids, which varies in the range of 5–10 nm to 50–200 nm [24–30], simulations are still resource-demanding. One way to address this challenge is to use coarse-grained (CG) molecular dynamics to replace several heavy atoms with large beads. A study by Clulow et al*.* using the CG methodology showed good agreement between the experimental and computational data [31]; specifically, colloidal structures with ellipsoidal shapes were observed using both MD and SAXS. Nevertheless, a discrepancy in specific shapes was observed, which was attributed to the lack of explicit hydrogen bonds between molecules in the micelles. Therefore, although CG MD models allow for the simulation of larger systems on longer timescales, they are inherently less accurate than all-atom simulations. Unless careful validation is performed, errors arising from a lack of accuracy can lead to meaningless simulation results.

An alternative approach to address the high computational cost of estimating free energy profiles, which is not dependent on the choice of all-atom vs. CG resolution, is to evaluate the free energy changes at specific points of interest along the reaction coordinate [32]. For example, these points can be the center of a micelle, at the free energy minimum (the most populated position in an unbiased, equilibrated simulation), and out in the bulk. Such calculations can be performed using one of several free energy calculation methods, such as free energy perturbation theory, Bennet acceptance ratio, or thermodynamic integration [11, 33]. The small molecule can either be gradually inserted within the micelle or, following initial insertion, be gradually deleted in several steps [34–36]. Interpolation between such point calculations is a somewhat common practice, mostly in earlier research performed with MD [37, 38], and could come into demand again, for example, when using MD simulations for extensive screening as part of an ML workflow. One challenge associated with this approach is the unknown complexity of the free energy profile, with several potential wells and barriers along the reaction coordinate.

This study explored the potential of the aforementioned approaches in evaluating the solubilization capacity of intestinal fluid micelles for four small molecules. The micelles are composed of either bile salts, represented by sodium taurocholate (NaTC), or phospholipids, represented by 1,2-dilinoleoyl-sn-glycero-3-phosphocholine (DLiPC), or a combination of both. Three drug molecules with low aqueous solubility were selected: prednisolone (logP = 1.6 [39, 40]), danazol (logP = 4.2 [41]), and probucol (logP = 10.91 [42, 43]). As reported by Parrow et al*.,* the affinity of drugs for micelles in intestinal fluid is governed by the hydrophobicity of the compounds [44]. In addition, sodium caprate was chosen as an example surfactant. It is an amphiphilic molecule that can be included in pharmaceutical dosage forms as a transient permeability enhancer for macromolecular drugs. All three drugs are neutral compounds with molecular weights comparable to sodium taurocholate.

**Fig. 1:**
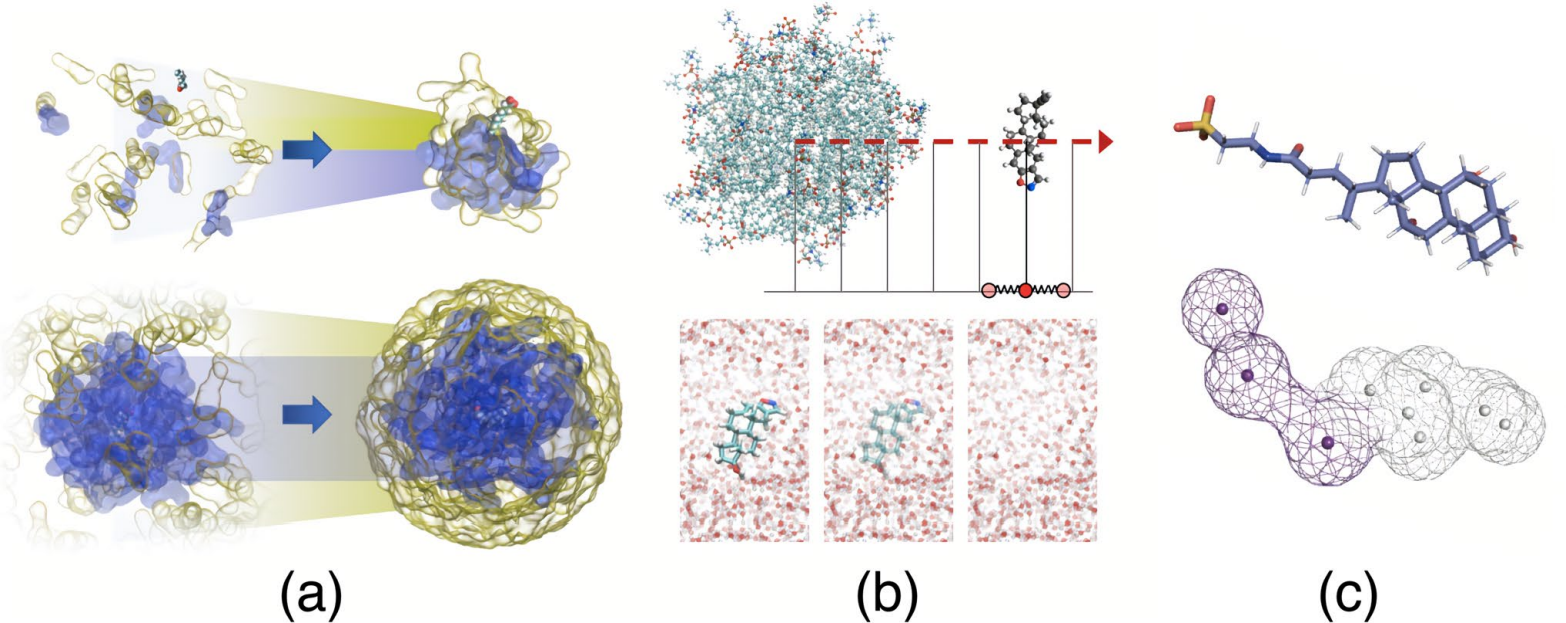
Schematic representation of the methods used in this study. **a** Colloids self-assembled from randomly placed molecules (upper part of the panel) are compared to those with a fixed phospholipid core (lower part); **b** umbrella sampling simulations providing full free energy profiles are compared to point-wise free-energy calculations; **c** all-atom simulations are compared to coarse-grained alternatives

Four methods were compared using different example systems to examine their applicability to extensive FE profile screening (see the graphical summary of the methods in Fig. 1). In the results section, the data are presented as follows: first, in “Aggregation, drug loading” section, the aggregation of the components and the formation mechanisms of unbiased micelles are described; in “Micelle composition” section, all-atom MD free energy profiles and radial distribution functions for the four compounds as they interact with the freely assembled micelles of different compositions are analyzed; in “Micelle organization comparison” section, free energy profiles between the randomly aggregated micelles and predefined ones are compared; analysis of the interpolated free energy profiles, based on several values computed with multistate Bennett acceptance ratio (MBAR), is presented in “Umbrella sampling vs point-based free energy calculation” section; finally, the coarse-grained simulations results are compared with the corresponding profiles of the freely assembled colloids at all-atom resolution. In this study, we analyzed how the specificity of the simulation protocols can be optimally used to run an extensive series of simulations for micelle solubilization screening.

## Materials and methods

### General molecular dynamics set-up

The all-atom molecular dynamics simulations were performed with GROMACS version 2018 [45–47] using the Generalized Amber [48, 49] and Slipids force fields [50–53], with the TIP3P water model [54]. A universal cut-off of 1.2 nm was used for both electrostatic and Lennard–Jones interactions. Long-range electrostatic interactions were calculated using the Particle Mesh Ewald (PME) method [55, 56]. Canonical (NVT) and isothermal-isobaric (NPT) ensembles were used at the equilibration stages, and the latter was used for the production runs. The temperature was fixed at 310 K using a Nose–Hoover thermostat with the time constant set to $\tau =1$ ps. A time step of 2 fs was used for all production simulations. A Parrinello-Rahman barostat was used for isotropic pressure coupling (${P}_{ref}=1$ atm, ${\tau }_{P}=2$ ps). Covalent bonds were constrained using the P-LINCS algorithm. Topologies for all-atom representations of danazol, sodium caprate, probucol, prednisolone, DLiPC, and NaTC molecules were produced using the Stage software [57]. Partial charges were derived using the PyRed server [58].

The Martini force field was used for the CG simulations [59, 60]. The topologies for coarse-grained molecules were obtained from previous studies by Hossain et al*.*, Parrow et al*.,* and Clulow et al*.* [31, 44, 61]. The integration step was gradually increased from 0.001 to 0.03 fs in a series of equilibration simulations with a total duration of more than 300 ns. A Berendsen thermostat and barostat were used during the equilibration stage; however, the Nose–Hoover thermostat and Parrinello-Rahman barostat were used for temperature and pressure coupling (${\tau }_{T}=4$ ps, ${\tau }_{P}=4$ ps, ${T}_{ref}=310$ K, ${P}_{ref}=1$ atm, compressibility = $3 \times {10}^{-4}$) for the production runs. The cut-off radii for the van der Waals and Coulomb interactions were set to 1.1 nm.

The simulation boxes were assembled using the Packmol software [62], either randomly or in a partially predefined manner (In the CG simulations, the initial placement of all molecules was random). In the case of predefined micelles, the phospholipidic core of the micelle was organized in a spherical shape, with the hydrophilic headgroups restricted to an outer shell (more than 3.1 nm from the center of the sphere) and the lipid tails pointing inward (between 0.6 and 0.8 nm away from the center). The bile salt molecules were distributed close to the PL core and adsorbed on the micelle surface during equilibration, forming a core–shell PL-BS colloid. Following equilibration, the bile salt and phospholipid molecules were restricted with a flat-bottom potential using a force constant ${F}_{fb}=\mathrm{10,000}$ kJ ${mol}^{-1}{nm}^{-2}$ to prevent atoms from moving further than 0.2 nm from the predefined position. The randomized systems were equilibrated with respect to the total energy and aggregation number of the colloids. The systems were considered to be equilibrated when a close association between the colloid and free bile salt or lipid molecules did not result in further aggregation. At this stage, any remaining non-aggregated bile salts and phospholipids were removed and replaced with water. The small solute molecules were added to the bulk after colloid formation. In all four cases, the solutes adhered to the micelles. The final configuration from the equilibration stage trajectory was used to run a pulling simulation for enhanced sampling. The sizes of the simulation boxes are listed in Table 1. To satisfy the minimum image convention after aggregation equilibration, the box size was always chosen such that the diameter of the micelle was at least two times smaller than the smallest box side length.

**Table 1:** Composition of the simulation boxes and characteristics of the observed colloids

	Micelle	Molecule	Box composition			${R}_{g}$*	$e$	Box
			DLiPC	NaTC	W			Length*
Self-assembled	BS	S. caprate	–	8	8000	0.9 ± 0.04	0.374	6.26
		Danazol	–	8	8000	1.03 ± 0.03	0.23	6.26
		Prednisolone	–	8	8000	1.03 ± 0.02	0.28	6.26
		Probucol	–	8	8000	1.12 ± 0.02	0.4	6.26
	PL	S. caprate	14	–	15000	1.4 ± 0.02	0.117	7.24
		Danazol	14	–	15000	1.4 ± 0.02	0.118	7.23
		Prednisolone	14	–	15000	1.4 ± 0.02	0.133	7.23
		Probucol	14	–	15000	1.41 + 0.02	0.122	7.23
	BS-PL	S.caprate	6	23	25000	1.55 ± 0.01	0.261	8.53
		Danazol	5	25	25000	1.58 ± 0.01	0.183	8.53
		Prednisolone	3	11	25000	1.24 ± 0.01	0.126	8.98
		Probucol	3	16	25000	1.4 ± 0.01	0.12	8.49
Pre-organized	PL	S. caprate	48	–	25000	2.11 ± 0.01	0.065	9.31
		Danazol	48	–	25000	2.01 ± 0.01	0.097	8.65
		Prednisolone	48	–	25000	2.14 ± 0.01	0.099	9.33
		Probucol	48	–	25000	2.15 ± 0.01	0.073	9.32
	BS-PL	S. caprate	40	160	65000	3.06 ± 0.02	0.053	12.8
		Danazol	40	160	65000	2.97 ± 0.01	0.088	12.87
		Prednisolone	40	160	65000	2.94 ± 0.01	0.034	12.15
		Probucol	40	160	65000	2.95 ± 0.01	0.05	12.17

${{\text{R}}}_{{\text{g}}}$**—**gyration radius, ${\text{e}}$—eccentricity. The simulation boxes were cubic in all cases. ${{\text{R}}}_{{\text{g}}}$ is presented as the average value ± standard deviation

*DLiPC* 1,2-dilinoleoyl-sn-glycero-3-phosphocholine, *NaTC* sodium taurocholate, *W* water, *S. caprate* sodium caprate. An eccentricity value of zero indicates a perfectly spherical shape, whereas a higher value corresponds to a more elongated or irregular shape

^*^in nm

In another series of simulations, we compared the randomly self-assembled micelles with preorganized micelles. In the latter model, phospholipid tails occupy the core of the colloid, whereas BS resides in the outermost shell. With this preparation, the micelles were uniform in all directions to enable comparison between various small molecules pulled from the colloid center of mass to the bulk.

For randomly assembled micelles, the aggregation numbers were obtained from the simulations by simple counting. In contrast, the number of DLiPC molecules for the predefined micelles was chosen to completely screen the small molecule in the center from contact with water. The number of BS molecules was then taken to be four times higher than that of the PL, in accordance with the 4:1 ratio discussed above. Phospholipids tend to aggregate into lamellar structures rather than micelles in the absence of other components such as bile. However, at low concentrations, aggregation can lead to micelle formation. Thus, we assumed that pure PL micelles were present in the intestinal fluid.

Micelle eccentricity was calculated as

1 $$e = 1 - I_{min} /I_{aver} ,$$

where ${I}_{min}$ is the smallest moment of inertia along the x-, y-, and z-axes, and ${I}_{aver}$ is the average of all three moments of inertia [63]. Eccentricity calculations were performed over the last 10 ns of the equilibrated system simulations.

The solvent accessible surface analysis (SASA) was done to compare the area of the APIs exposed to water in AA and CG simulations and was calculated via the “measure sasa” command in VMD. A probe size of 0.26 nm was used to facilitate a fair comparison between AA and CG (a “standard” probe size for atomistic simulations is otherwise 0.14 nm). As a sensitivity analysis, we also used an even larger probe size (0.4 nm) to check whether the results would be qualitatively unaffected. The SASA of the aqueous interface of the APIs was divided by the total area of the molecules to evaluate the percentage of the surface in contact with water molecules.

### Umbrella sampling parameters

**Fig. 2:**
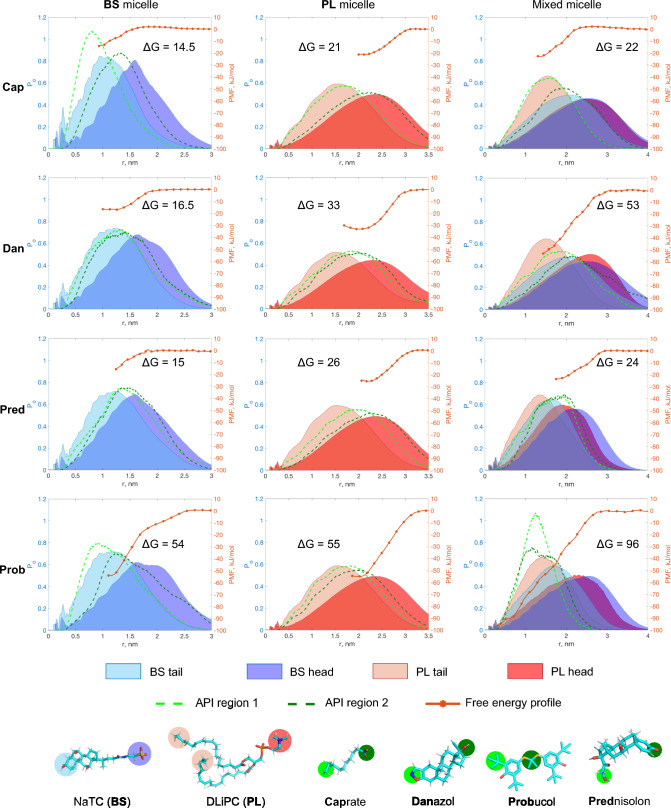
Combined free energy profiles and radial distribution of select beads. Free energy differences ($\Delta G$) are expressed in kJ/mol. The positions of various groups of the small molecules are depicted in dark and light green and are mapped in the lowest panel

**Fig. 3:**
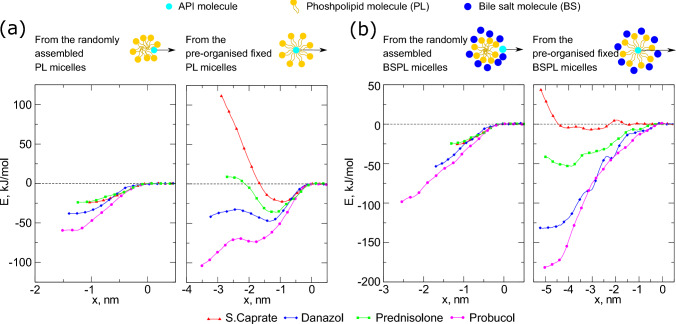
Comparison of free energy profiles of self-assembled and pre-organized PL and BS-PL colloids. The top panel shows a schematic representation of the steered MD simulations, in which the small molecules were pulled from the initial position, either at the center of the pre-organized micelle or at the surface of the self-assembled aggregates

**Fig. 4:**
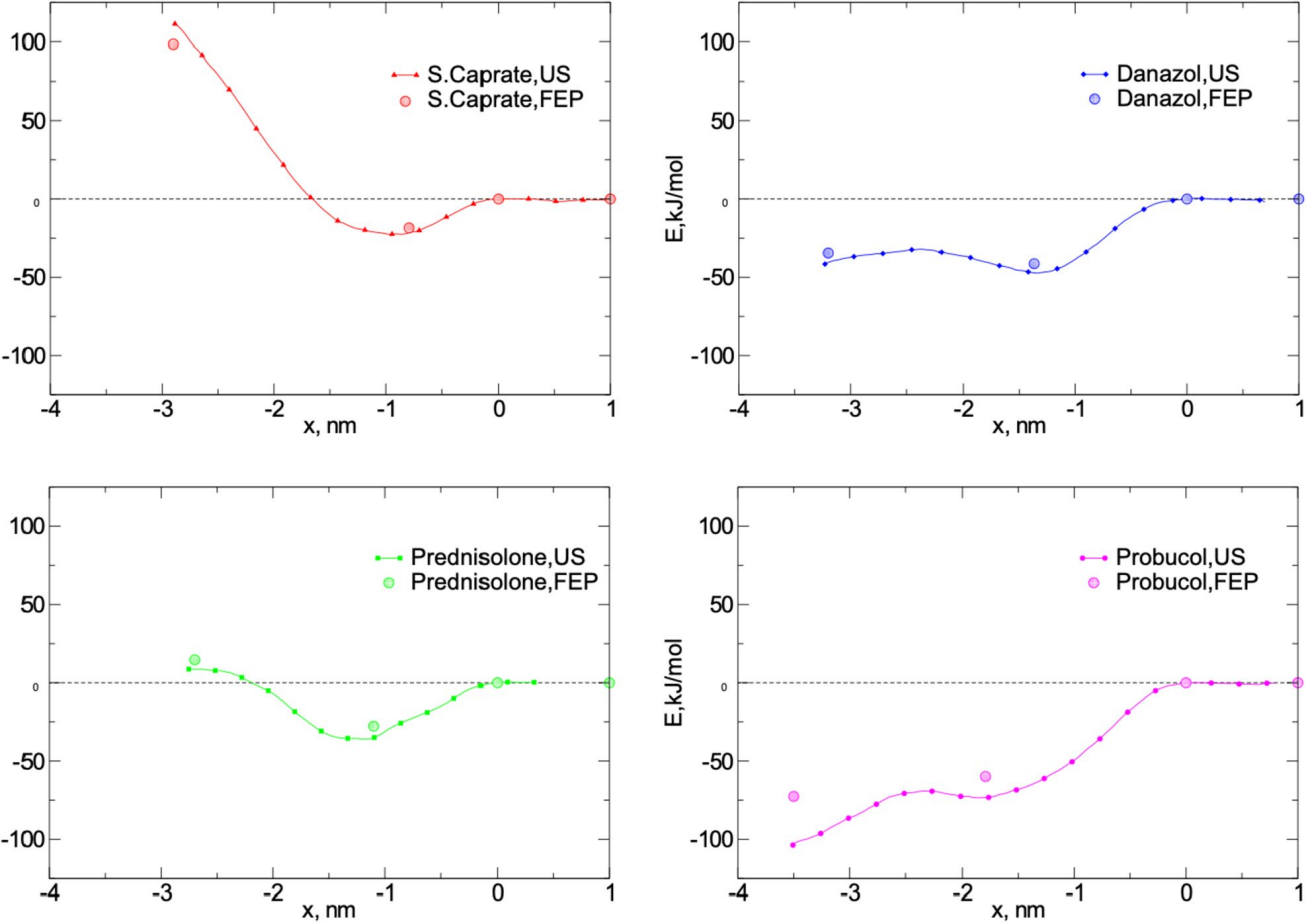
Mapping of the selected FE point (FEP) calculations in the bulk, at the interface, and in the center of the pre-organized PL colloids onto the corresponding umbrella sampling (US) profiles. The profiles were normalized to the free energy change in bulk. Four points are shown, with the bulk being depicted with two points: right at the interface and at a distance of one nm from it in the bulk. Standard deviations of the FE calculations do not exceed the size of the corresponding markers

**Fig. 5:**
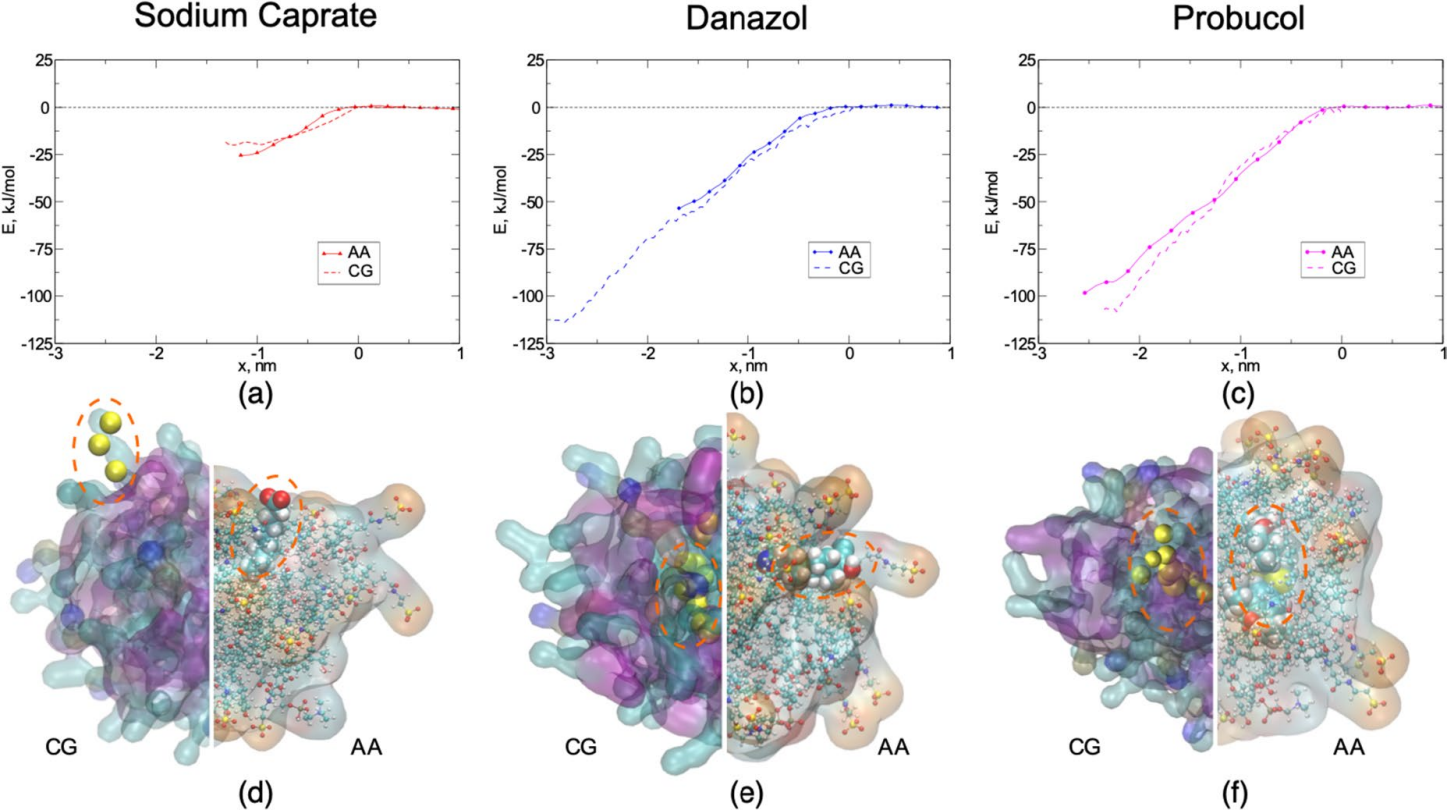
Comparison of the coarse-grained (CG) and all-atom (AA) model profiles for sodium caprate (**a**), danazol (**b**), and probucol (**c**). The combined CG and AA visual representations of the micelles and the equilibrated position of the API within (marked with dashed red ellipsoids), **d**, **e**, and **f**, respectively. In the CG representation, the danazol molecule is completely covered, whereas in AA it has contact with water molecules

Free energy ($\Delta G$) profiles were obtained using the US method [14, 64]. First, each small molecule (danazol, sodium caprate, probucol, and prednisolone) was slowly pulled from its initial position in each micelle (BS, PL, or BS-PL, and from either the center or from the position observed during an unbiased run). Each molecule was pulled beyond the point where no contact with the micelle remained, and snapshots separated by 0.05 nm along the pull coordinate were extracted These trajectory windows were then used to generate a free energy profile via the weighted histogram analysis method (WHAM) [65]. The molecule was restricted to each window with a harmonic potential, and simulations were run for each window (20 ns for all-atom, and 90 ns for the CG resolution). To hold the molecules within the less energetically favorable windows to enable adequate sampling, the force constants in the all-atom simulations were increased to 25,000 kJ mol^−1^ nm^−2^ after manual inspection of the WHAM histogram overlap. Bootstrap analysis was employed to statistically analyze the free energy profiles, providing robust estimates of uncertainty and variability in the calculated values (Figs. 1–5 in Online Appendix A). In the coarse-grained simulations, the force constants were generally lower because the energy barriers were smoothed by averaging the degrees of freedom of the molecules. WHAM implemented in Gromacs was used for analysis with the command gmx wham [66].

The Jacobian correction was applied to the free energy profiles to remove the artificial decrease in $\Delta G$ at longer distances [16, 67]. This decrease occurs because the integration volume increases for each consecutive bin from the micelle center. The equation used to modify the energy values is

2 $$\Delta G\left(r\right)= \Delta {G}_{WHAM}\left(r\right)+2 {k}_{B}T {\text{ln}}r$$

where $\Delta {G}_{WHAM}$ is the original profile, ${k}_{B}$ is the Boltzmann constant, $T$ is the temperature, and r is the distance from the micelle center of mass.

### Point-wise free energy calculations

To perform free energy calculations at certain points within a micelle, we used a PL micelle with a predefined spherical structure. To keep the molecule fixed at a particular position, the Gromacs pull code was applied. MBAR [68] was then used to calculate the free energy at three positions (the center of the micelle, at the equilibrium position within the micelle following diffusion from the aqueous bulk, and in the water phase) of the small molecule. The interactions with the micelle were decoupled in a procedure consisting of 20 intermediate steps (lambda states), where first the van der Waals interactions were gradually turned off, followed by Coulomb forces. The relative free energy differences for each lambda window were analyzed to ensure that the sampling was sufficient.

## Results

### Aggregation, drug loading

For the first part of the study, we used micelles self-assembled from BS, PL, or a combination of the two in a 4:1 ratio. The motivation was to understand the individual contributions of these two major components of the intestinal fluid to the solubilization of each of the four molecules as well as any synergetic effects. Initially, all the systems contained more molecules than those specified in Table 1. By monitoring the equilibration of micelle aggregation kinetics, it was found that the aggregation number of NaTC molecules in a BS-only micelle was approximately eight, and the number of DLiPC molecules stably residing in a PL micelle was approximately 14.

When both bile salts and phospholipids were present at the beginning of the equilibration process, the resulting micelles contained approximately 24 NaTC and six DLiPC molecules (i.e., the 4:1 ratio was maintained upon micelle formation in the simulations). In addition, the exchange of individual NaTC and DLiPC molecules continued even after 100 ns; however, the ratio in the micelles did not change significantly. The micelles were prolate ellipsoids with eccentricity values between 0.12 and 0.4 (see Table 1). Pure PL aggregates were generally more spherical, whereas the BS aggregates were the least spherical.

### Micelle composition

The number of molecules and sizes of the self-assembled PL and mixed BS-PL micelles were significantly larger than those of BS-only micelles. The average gyration radii of the mixed BS-PL self-assembled micelles were found to be 1.5, 1.4 nm, and 1.02 nm. Owing to the hydrophobic effect during the self-assembly process, the tails of the DLiPC molecules tended to aggregate at the center of the micelles to minimize contact with water. Sodium taurocholate molecules are more complex in terms of lipophilicity, with multiple sites across the molecules containing hydrophobic and hydrophilic groups. This makes them prefer the surface of the aggregate, creating a shell around the DLiPC core, with the DLiPC headgroups oriented towards the outside of the micelle. Coalescence events between individual micelles were also observed during the simulations; however, these aggregates would split into smaller colloids within nanoseconds.

As discussed above, the inhomogeneous distribution of the BS and PL components makes it difficult to compare the free energy profiles of different molecules because the local environment might differ during pulling (and US simulations) for each compound. Moreover, the shape of the micelles may be affected by the small drug or surfactant molecule. For example, sodium caprate (a fatty acid) has a hydrophilic head in contact with water in the randomly assembled micelles. Its interactions with the colloid are, to a great extent, dependent on the coverage of the hydrophobic tail by the micelle molecules. In the case of the BS-only aggregate, this tail was only partially inserted into the micelle. The free energy of a sodium caprate at the BS micelle surface was 14.5 kJ/mol lower than that of the bulk (Fig. 2). In the PL micelle, the free energy difference between the surface and bulk is ~ 21 kJ/mol, which is approximately equal to that in the mixed BS-PL micelle case, indicating no obvious synergetic effect for a mixed BS and PL micelle for sodium caprate. In contrast, danazol was solubilized twice as well in PL compared to BS micelles, but even better in the mixed micelle. The total effect of the mixed micelle on the solubilization capacity of danazol is stronger than the sum of the individual contributions of NaTC and DLiPC (~ 53 kJ/mol *vs* ~ 16.5 kJ/mol and 33 kJ/mol respectively). Thus, danazol tightly interacts with both BS and PL molecules and inserts almost completely into the mixed micelles. Prednisolone is more solubilized by the phospholipid molecules. The change in the free energy when prednisolone was pulled from the mixed micelle was slightly lower than that for the smaller PL aggregate (~ 26 versus ~ 24 kJ/mol). The free energy of solubilization of prednisolone in the BS micelle is of the same order as that of sodium caprate (~ 15 kJ/mol). Finally, probucol, the most hydrophobic compound, is almost equally well solubilized within BS and PL micelles (− 54 kJ/mol and − 55 kJ/mol), and in the mixed micelle, the free energy is almost equal to the sum of the two (− 96 kJ/mol). The drug molecule is in the most favorable energy state once it is completely screened from contact with water.

From the results above, it seems that both danazol and probucol are better solubilized in the micelles than prednisolone and sodium caprate.

Prednisolone solubilization in PL aggregates seems to be more advantageous compared to BS micelles. Danazol resides on the surface of the colloid upon approaching the membrane, and it is possible that the molecule would transfer to the membrane. Probucol was well-solubilized by both BS and PL micelles without significant differences in affinity. However, as probucol is entirely covered with NaTC and DLiPC molecules, any transfer from the micelle to the cell membrane towards systemic circulation would require either breakage of the micelle or fusion between it and the bilayer. In addition, because the results suggest that a probucol molecule would prefer to occupy the center of the micelle, aggregation, and as a consequence, crystallization might potentially occur within the colloid.

Free energy profiles obtained in this way for colloids randomly assembled around a small molecule thus provide valuable information, but they might be insufficient if energy barriers are present on the way from the bulk to the FE minimum state. This may be a more realistic scenario for drug molecules released from a dosage form in the small intestine. Thus, a profile spanning from the center of the colloid to the bulk may be needed to verify the properties of the free energy well within the colloid.

### Micelle organization comparison

Simulations with preorganized micellular structures were performed for pure phospholipids and mixed BS-PL micelles. We were unable to construct a preassembled BS micelle, as those colloids tended to fall apart during the initial simulation phases. A substantial increase in the size of the colloids (Table 1) was necessary to ensure symmetry with respect to the molecular orientation and composition of the colloid.

The free energy profiles were compared for both types of micelles. The free energy profiles of the self-assembled and pre-organized pure PL micelles were qualitatively consistent (Fig. 3a). In all cases, except for sodium caprate, the energy wells were deeper in the larger, pre-organized micelles. For poorly soluble drugs, it makes sense that a larger colloid would screen drug molecules better from aqueous contact. Danazol and prednisolone occupied the PL headgroup region, whereas probucol moved to the center of the colloid after overcoming a small energy barrier at 2–2.5 nm from the interface with water.

For the BS-PL micelles, manual organization into separated PL and BS layers leads to differences in free energy compared to randomly assembled micelles (Fig. 3b). Although the rank order was the same, the energy minima for probucol and danazol were lower (178 kJ/mol and 129 kJ/mol compared to 99 kJ/mol and 51 kJ/mol, respectively), and the positions of the minima were also shifted. Probucol is solubilized to the highest degree, in the same way as for the self-assembled micelles, while caprate, on the other hand, does not show a significant free energy minimum at the outer layer of the mixed pre-organized micelle. There is also a potential barrier at a depth of 2 nm from the water-micelle interface, which indicates the loss of contact with water for the caprate head group.

### Umbrella sampling vs point-based free energy calculation

An alternative way to study micelle-drug interactions is to perform free energy calculations only at specific points of interest along the reaction coordinate. To investigate this approach, we used pre-organized micelles and determined the free energy at three different points: in the micelle center, at the unbiased simulation equilibrium position, and in the bulk (two points were placed on the graph for clarity, at zero and one nm away from the outermost contact with the micelle; only one measurement in bulk was made). As before, the zero point was set as a reference to correspond to the bulk water.

For all four small molecules, there was good (and somewhat expected) quantitative agreement between the free energy change values measured at the chosen points and the profiles observed from the US simulations (Fig. 4). The largest discrepancy was observed for probucol, where the values of $\Delta G$ were higher than those predicted by the US. Nevertheless, this approach seems to work well for the comparison of small molecule profiles. The limitation of the method, as compared to US simulations, is the lack of information on whether any intermediate energy barriers are present. For example, for danazol and probucol, no information can be obtained from the FE calculations about the region with small local free energy maxima between the FE points. This can be compensated, to some extent, by introducing more measurement points along the reaction coordinate.

### Methods comparison: AA–US vs CG–US

Another solution to speed up the simulations is to use coarse-grained resolution. Therefore, we tested how much faster the simulations are with CG compared to AA, while at the same time examining the differences in the results as a consequence of the reduced level of detail associated with coarse-graining.

The self-assembled BS-PL mixed micelles were chosen, to include interactions with both types of molecules, and also let the small drug/surfactant molecule freely find its optimal position within the micelle. The simulations in this section focused on sodium caprate, danazol, and probucol, and the resulting free energy profiles observed from the CG US simulations can be seen in Fig. 5.

Sodium caprate and probucol showed a good match between AA and CG profiles. However, in the danazol case, the results appeared to be significantly different between AA and CG, both about the position and depth of the energy minimum. The energy minimum for danazol in the AA simulations is ~ 51 kJ/mol, with the position being approximately 1.65 nm from the surface of the water. The corresponding free energy change for the CG simulations is more than double that, 112 kJ/mol, and the position is deeper at a distance of 2.8 nm from the micelle surface.

To further understand the AA and CG behavior, we analyzed the SASA of the APIs while in their equilibrium position in the micelle (specifically, only the surface exposed to water was calculated) using two probe sizes, 0.26 nm and 0.4 nm (Table 2). The ratio of this surface exposed SASA to the total surface area of the API was then calculated to find out the portion of the drug surface in contact with water. The data showed an expected trend between the APIs. The contact area with water was minimal for probucol, the most hydrophobic drug, covering 0–4.4% of the surface. Sodium caprate, as a surfactant, had the highest percentage, between 15.6–31.6% of its surface area in contact with water. Danazol had 3.8–4.2% of its surface at the interface with the aqueous phase. Interestingly, the SASA differences between the all-atom and coarse-grained simulations were the least pronounced for danazol, the API that had the most different free energy profiles between the AA and CG resolutions (Fig. 5). This illustrates the complexity of the interplay between the model molecules and the validation required to perform reasonable simulations.

**Table 2:** Percentage of surface area for the small molecules in contact with water molecules, as observed in all-atom (AA) and coarse-grained (CG) simulations

Probe size	Model	S.caprate	Danazol	Probucol
0.26 nm	AA	20.9 ± 5.9	7.4 ± 3.2	0
	CG	31.6 ± 12.0	8.2 ± 7.1	4.4 ± 7.3
0.4 nm	AA	15.6 ± 5.11	3.8 ± 2.8	0
	CG	20.4 ± 10.4	4.2 ± 4.36	2.3 ± 4.8

Solvent accessible surface area (SASA) analysis was performed for the area calculation with probes of 0.26 nm and 0.4 nm for both AA and CG simulations. ${\text{SASA}}$ is presented as the average value ± standard deviation

CG simulations have significantly higher performance, but extensive validation, beyond octanol–water partitioning might be required. One of the possibilities might be the combination of the AA and CG techniques, where key points from the AA simulations can serve as a check for the CG free energy profiles. Alternatively, the CG free energy profile can be used as an approximate map or reference for the targeted point-wise free energy calculations.

The computational time required to run the simulations varies significantly and depends on hardware, software, and simulation parameters. However, two characteristics can provide an estimate of the computational costs for simulations with different protocols described in this study. One is the number of windows required to determine the free energy profile, and another is the average time for running such a window. We summarised these data in Table 1 in Online Appendix A. As can be seen, CG and FEP approaches are more efficient. Moreover, multiple screenings might be required with analysis time, as the spring constant for umbrella sampling needs to be gradually adjusted over several iterations, covering the entire space along the reaction coordinate. Nevertheless, CG mapping and validation might be a time-consuming procedure too. Even though several automated tools for topology building are available, it is highly recommended to ensure the validity of the molecules and fine-tune them in many cases. Therefore, fast screening might be organized with scripts and little validation, but a more thorough analysis would be required to introduce the accurate CG topologies. Table S1 is only shown to give an estimate of the time required to run simulations with different protocols used in this study. As can be seen from the last row, one the hardware we used, one can run a complete series of AA simulations for self-assembled colloids of mixed BS and PL in 48 h, whereas the corresponding CG series can be finished within 8.8 h. FE point calculations can be finished within 5.3 h for three points (API in water, outmost energy well, and in the center of the colloid). However, for the later approach, CG simulations should be first run to define the approximate position of the energy well.

## Discussion

Bile salts and phospholipids play important roles in solubilization of small molecules in the intestine. Model intestinal fluids, such as FaSSIF, are very useful but not always able to fully represent the colloidal structures present in human intestinal fluids [69]. With the ratio of the two major components (bile salts and phospholipids) maintained close to 4:1, it is of interest to study the interactions of small drug molecules with colloids in a broad range of observed colloidal sizes [24, 26]. In a study by Elvang et al*.* asymmetrical flow field-flow fractionation (AF4) and multi-angle laser light scattering (MALLS) allowed the detection of even smaller pure bile salt aggregates with a diameter of 2–3 nm [70]. This agrees well with a model proposed by Carey and Small and later refined by Mazer et al.: bile salts and phospholipids are likely to form mixed discs of various sizes, starting from several molecules. The side rim of the disks is formed by bile salts, whereas in the central part, phospholipids would form a cylinder-shaped bilayer [71, 72].

In this theoretical study, we evaluated the capacity of several MD protocols to predict the effects of BS and PL solubilization of small molecules for different pharmaceutical purposes. Such protocols can be useful for qualitative comparison of solubilization in intestinal colloids of specific structures. Two important factors to consider when choosing a specific protocol for extensive studies are the accuracy of the model and its affordability. Here, we attempt to generate insights into the optimal approach by performing several comparisons.

First, free energy profiles provide valuable information for structured studies on the interactions between APIs and the components of the intestinal colloids. Danazol and probucol were better solubilized in mixed BS-PL colloids, whereas the free energy changes associated with the removal of prednisolone molecules from PL and BS-PL colloids did not differ significantly. Notably, good solubilization does not necessarily imply efficient drug delivery. The aggregation of multiple APIs within a colloid can potentially result in nucleation and re-crystallization. Moreover, the release of the drug may be hindered by the incorporation of the API deep inside the colloid. On the other hand, as shown in an MD study on danazol, small BS-PL micelles can both deliver molecules to the surface of the membrane and fuse into the membrane together with the drug [21]. The free energy change associated with the danazol molecule being pulled from the 1-palmitoyl-2-oleoyl-sn-glycero-3-phosphocholine (POPC) membrane was higher than that from the BS-PL micelle in our study (60 kJ/mol vs 53 kJ/mol, respectively), indicating that the drug would likely be eventually released from the colloid to the membrane. Self-aggregation of the APIs within the micelles may also be insufficient for recrystallization. In a study by Edueng et al*.* clustering of probucol molecules in an amorphous solid dispersion did not lead to crystal formation [73]. Thus, multiple factors together define the fate of a drug solubilized in intestinal fluid colloids, many of which can potentially be addressed with MD simulations.

The free energy profile of API-micelle interactions is one of the main tools that can be extensively used for the design of novel drug delivery systems. However, inhomogeneities and colloid asymmetry can cause convergence issues and require vast computational resources to be effectively addressed. In this study, we demonstrated that it is possible to use a less variable system, where the structure of the colloid is strictly defined. However, it comes at the cost of some inaccuracies in the representation of the colloid. In the case of PL micelles, the free energy profiles are in good qualitative agreement in the region from the bulk phase to the bottom of the potential well at 0.8–1.5 nm from the surface of the micelle. Thus, pulling the drug molecule from the surface of the pre-organized PL micelles would result in a profile very similar to that of a self-assembled PL colloid. At the same time, as can be seen from the example of probucol, the energy minimum might not be reached when the molecule comes from the bulk and faces the barrier behind the potential well (Fig. 3b, to the left from 2 nm). The height of the barrier is also not completely clear from the free energy calculations of the freely-assembled system. We performed tests in which the molecule was pulled towards the center of the self-assembled colloid, but it was found inconclusive, as a “tail” of water molecules follows the drug/surfactant molecule. This leads either to breakage of the aggregate or, if the molecules are restrained, to an increase in $\Delta G$ to unrealistically high values (100–200 kJ/mol in the center; data is available on request). A situation where the drug molecule can be screened from aqueous contacts is also rather theoretical, since, if the aggregation number is close to 14, a micelle with 48 DLiPC chains might be unrealistic. Nevertheless, such a profile provides insight not only into the free energy associated with the adsorption on the surface of the colloid but also into potential deeper levels of permeation and corresponding energy changes. In addition, conclusions on the nature of solubilization can be drawn from the profiles. For example, sodium caprate has a low affinity for DLiPC tails, whereas it is more energetically favorable to cover the hydrophobic tails within the upper layers of the micelle. From the randomly assembled micelle-free energy profile, it can be seen that sodium caprate has affinity to the micelle, and the first potential well can be observed at a distance of 1 nm from complete water coverage. Overall, the pre-defined colloidal structure approach might be useful for extensive and rough pre-screening or comparison of drug-micelle interactions.

Point-wise free energy calculation is another approach to prescreen drug solubilization. It can provide a reasonable approximation of the more informative and resource-demanding calculations with a significantly lower number of CPU hours used. Here, we only performed three measurements, in the colloid center, in the bulk, and in the unbiased simulation equilibrium position, when the API approached the micelle from the bulk. However, in this protocol, energy barriers and wells in the intervals between the points of measurement could potentially be missed. To address this, additional points can be added between the existing ones, with for example the golden-section search, until the FE function is continuous and smooth. Another alternative is to perform this with the help of an approximate profile coming from other simulations, such as coarse-grained enhanced sampling simulations. We would then know the approximate positions of the energy wells along the reaction coordinate, but those might not match completely between the AA and CG models [74]. Point-wise FE calculations might be specifically useful for generating input data for ML since the solvation energies at these points alone can constitute part of a molecular formulation fingerprint. Molecular fingerprints would encompass the physical and chemical properties of the APIs, various energy terms as well as the interactions with gastrointestinal fluids and cell plasma membrane. The later inputs can be gained from FE calculations of APIs at different position with respect to colloids and membranes. Such dataset provides a comprehensive and detailed insight into how the drugs interact with biological environments. By harnessing the principles of ML, one can potentially extrapolate from these measurements to predict a range of critical pharmacological properties, such as solubility, permeability and drug delivery.

CG models can provide the most efficient combination of reasonable accuracy and realistic micellular organization (achieved via self-assembly of the molecules), but as we discovered in this study, the differences in solubilization patterns can be substantial. If the balance between computational expenses and accuracy is a key factor of the workflow, combining several techniques might be the optimal solution. In certain large-scale multicomponent studies, a combination of a unified colloid and CG pre-screening followed by point-wise AA measurements of the free energy could be beneficial. At the same time, if computational cost is not the limiting factor, a thorough analysis considering all degrees of freedom, including asymmetry of the colloids and rotational sampling of the API, would in many cases provide more accurate information. Martini 3, a newer version of the coarse-grained force field used in this study, is capable of introducing more flexibility and accuracy, while still significantly reducing the computational costs [75]. The balance between the convergence and computational cost can be optimized dynamically throughout simulations [76].

Given the rapid development of hardware, efficiency of simulations and machine learning processes will grow. At the same time, better tools for automatic mapping of the AA topologies onto CG will make such computational protocols even more efficient and applicable to predict the efficiency of drug delivery processes.

## Conclusions

Our study demonstrated that specific methods are preferable for specific purposes. Selecting the optimal combination can effectively reduce computational costs while maintaining accuracy. Overall, all reported methods showed good agreement; however, some notable exceptions emerged, such as the difference between the free energy profiles of a danazol molecule pulled from a BS-PL colloid in all-atom (AA) and coarse-grained (CG) MD simulations. Therefore, caution should be exercised when alternative methods are applied to replace AA umbrella sampling (US) simulations. In addition to coarse-graining, reconstructing the entire profile from several points could prove to be a valuable tool for minimizing computational costs. Moreover, the two methods can be combined to predict an approximate profile first and then improve the quality with more accurate AA simulations. Once automated, these approaches can expedite prescreening and qualitative comparison of free energy profiles, offering a faster alternative to other methods.

## Supplementary Information

Below is the link to the electronic supplementary material.

Supplementary file1 (DOCX 471 KB)

## Data Availability

The datasets generated during the current study are available from the corresponding author on reasonable request.

## References

[1] Bates TR, Gibaldi M, Kanig JL (1966) Solubilizing properties of bile salt solutions II. Effect of inorganic electrolyte, lipids, and a mixed bile salt system on solubilization of glutethimide, griseofulvin, and hexestrol. J Pharm Sci 55:901–906 5918525 10.1002/jps.2600550906

[2] Kossena GA, Boyd BJ, Porter CJH, Charman WN (2003) Separation and characterization of the colloidal phases produced on digestion of common formulation lipids and assessment of their impact on the apparent solubility of selected poorly water-soluble drugs. J Pharm Sci 92:634–648 12587125 10.1002/jps.10329

[3] Kossena GA, Charman WN, Boyd BJ, Porter CJH (2005) Influence of the intermediate digestion phases of common formulation lipids on the absorption of a poorly water-soluble drug. J Pharm Sci 94:481–492 15619248 10.1002/jps.20260

[4] Bates TR, Lin S, Gibaldi M (1967) Solubilization and rate of dissolution of drugs in the presence of physiologic concentrations of lysolecithin. J Pharm Sci 56:1492–1495 6060594 10.1002/jps.2600561123

[5] Rosoff M, Serajuddin ATM (1980) Solubilization of diazepam in bile salts and in sodium cholate-lecithin-water phases. Int J Pharm 6:137–146

[6] Dressman JB, Amidon GL, Reppas C, Shah VP (1998) Dissolution testing as a prognostic tool for oral drug absorption: immediate release dosage forms. Pharm Res 15:11–22. 10.1023/A:1011984216775 9487541 10.1023/a:1011984216775

[7] Jantratid E, Janssen N, Reppas C, Dressman JB (2008) Dissolution media simulating conditions in the proximal human gastrointestinal tract: an update. Pharm Res 25:1663 18404251 10.1007/s11095-008-9569-4

[8] Galia E, Nicolaides E, Hörter D et al (1998) Evaluation of various dissolution media for predicting in vivo performance of class I and II drugs. Pharm Res 15:698–705 9619777 10.1023/a:1011910801212

[9] Psachoulias D, Vertzoni M, Butler J et al (2012) An in vitro methodology for forecasting luminal concentrations and precipitation of highly permeable lipophilic weak bases in the fasted upper small intestine. Pharm Res 29:3486–3498 22890986 10.1007/s11095-012-0844-z

[10] Riethorst D, Mols R, Duchateau G et al (2016) Characterization of human duodenal fluids in fasted and fed state conditions. J Pharm Sci 105:673–681. 10.1002/jps.24603 26228456 10.1002/jps.24603

[11] Hossain S, Kabedev A, Parrow A et al (2019) Molecular simulation as a computational pharmaceutics tool to predict drug solubility, solubilization processes and partitioning. Eur J Pharm Biopharm 137:46–55. 10.1016/j.ejpb.2019.02.007 30771454 10.1016/j.ejpb.2019.02.007 PMC6434319

[12] Zhang J, Tuguldur B, van der Spoel D (2015) Force field benchmark of organic liquids. 2. Gibbs energy of solvation. J Chem Inf Model 55:1192–1201. 10.1021/acs.jcim.5b00106 26010106 10.1021/acs.jcim.5b00106

[13] Liu S, Cao S, Hoang K et al (2016) Using MD simulations to calculate how solvents modulate solubility. J Chem Theory Comput 12:1930–1941. 10.1021/acs.jctc.5b00934 26878198 10.1021/acs.jctc.5b00934 PMC4945102

[14] Torrie GM, Valleau JP (1977) Nonphysical sampling distributions in Monte Carlo free-energy estimation: umbrella sampling. J Comput Phys 23:187–199

[15] Kästner J (2011) Umbrella sampling. WIREs computational molecular. Science 1:932–942. 10.1002/wcms.66

[16] Trzesniak D, Kunz A-PE, van Gunsteren WF (2007) A comparison of methods to compute the potential of mean force. ChemPhysChem 8:162–169. 10.1002/cphc.200600527 17131434 10.1002/cphc.200600527

[17] Gebhardt J, Kiesel M, Riniker S, Hansen N (2020) Combining molecular dynamics and machine learning to predict self-solvation free energies and limiting activity coefficients. J Chem Inf Model 60:5319–5330. 10.1021/acs.jcim.0c00479 32786697 10.1021/acs.jcim.0c00479

[18] Riniker S (2017) Molecular Dynamics Fingerprints (MDFP): machine learning from MD data to predict free-energy differences. J Chem Inf Model 57:726–741. 10.1021/acs.jcim.6b00778 28368113 10.1021/acs.jcim.6b00778

[19] Weinreich J, Browning NJ, von Lilienfeld OA (2021) Machine learning of free energies in chemical compound space using ensemble representations: reaching experimental uncertainty for solvation. J Chem Phys 154:134113 33832231 10.1063/5.0041548

[20] Yordanova D, Ritter E, Gerlach T et al (2017) Solute partitioning in micelles: combining molecular dynamics simulations, COSMOmic, and experiments. J Phys Chem B 121:5794–5809 28534622 10.1021/acs.jpcb.7b03147

[21] Kabedev A, Hossain S, Hubert M et al (2021) Molecular dynamics simulations reveal membrane interactions for poorly water-soluble drugs: impact of bile solubilization and drug aggregation. J Pharm Sci 110:176–185. 10.1016/j.xphs.2020.10.061 33152373 10.1016/j.xphs.2020.10.061

[22] Yuan F, Wang S, Larson RG (2015) Potentials of Mean force and escape times of surfactants from micelles and hydrophobic surfaces using molecular dynamics simulations. Langmuir 31:1336–1343. 10.1021/la5044393 25560633 10.1021/la5044393

[23] Turchi M, Kognole AA, Kumar A et al (2020) Predicting partition coefficients of neutral and charged solutes in the mixed SLES–fatty acid micellar system. J Phys Chem B 124:1653–1664. 10.1021/acs.jpcb.9b11199 31955574 10.1021/acs.jpcb.9b11199 PMC7060100

[24] Fatouros DG, Walrand I, Bergenstahl B, Müllertz A (2009) Colloidal structures in media simulating intestinal fed state conditions with and without lipolysis products. Pharm Res 26:361 19003522 10.1007/s11095-008-9750-9

[25] Riethorst D, Baatsen P, Remijn C et al (2016) An in-depth view into human intestinal fluid colloids: intersubject variability in relation to composition. Mol Pharm 13:3484–3493 27576295 10.1021/acs.molpharmaceut.6b00496

[26] Müllertz A, Fatouros DG, Smith JR et al (2012) Insights into intermediate phases of human intestinal fluids visualized by atomic force microscopy and cryo-transmission electron microscopy ex vivo. Mol Pharm 9:237–247 22136289 10.1021/mp200286x

[27] Walsh PL, Stellabott J, Nofsinger R et al (2017) Comparing dog and human intestinal fluids: implications on solubility and biopharmaceutical risk assessment. AAPS PharmSciTech 18:1408–1416 27600321 10.1208/s12249-016-0611-2

[28] Long MA, Kaler EW, Lee SP (1994) Structural characterization of the micelle-vesicle transition in lecithin-bile salt solutions. Biophys J 67:1733–1742. 10.1016/S0006-3495(94)80647-2 7819505 10.1016/S0006-3495(94)80647-2 PMC1225535

[29] Nawroth T, Buch P, Buch K et al (2011) Liposome Formation from bile salt-lipid micelles in the digestion and drug delivery model FaSSIFmod estimated by combined time-resolved neutron and dynamic light scattering. Mol Pharm 8:2162–2172. 10.1021/mp100296w 21988605 10.1021/mp100296w

[30] Müllertz A, Reppas C, Psachoulias D et al (2015) Structural features of colloidal species in the human fasted upper small intestine. J Pharm Pharmacol 67:486–492. 10.1111/jphp.12336 25580567 10.1111/jphp.12336

[31] Clulow AJ, Parrow A, Hawley A et al (2017) Characterization of solubilizing nanoaggregates present in different versions of simulated intestinal fluid. J Phys Chem B 121:10869–10881. 10.1021/acs.jpcb.7b08622 29090933 10.1021/acs.jpcb.7b08622 PMC6209315

[32] Tse CH, Comer J, Sang Chu SK et al (2019) Affordable membrane permeability calculations: permeation of short-chain alcohols through pure-lipid bilayers and a mammalian cell membrane. J Chem Theory Comput 15:2913–2924 30998342 10.1021/acs.jctc.9b00022

[33] Hansen N, van Gunsteren WF (2014) Practical aspects of free-energy calculations: a review. J Chem Theory Comput 10:2632–2647. 10.1021/ct500161f 26586503 10.1021/ct500161f

[34] Ben-Naim A, Marcus Y (1984) Solvation thermodynamics of nonionic solutes. J Chem Phys 81:2016–2027

[35] Shirts MR, Pitera JW, Swope WC, Pande VS (2003) Extremely precise free energy calculations of amino acid side chain analogs: comparison of common molecular mechanics force fields for proteins. J Chem Phys 119:5740–5761. 10.1063/1.1587119

[36] Klimovich PV, Shirts MR, Mobley DL (2015) Guidelines for the analysis of free energy calculations. J Comput Aided Mol Des 29:397–411. 10.1007/s10822-015-9840-9 25808134 10.1007/s10822-015-9840-9 PMC4420631

[37] Chipot C, Kollman PA, Pearlman DA (1996) Alternative approaches to potential of mean force calculations: free energy perturbation versus thermodynamic integration. Case study of some representative nonpolar interactions. J Comput Chem 17:1112–1131

[38] Matubayasi N, Liang KK, Nakahara M (2006) Free-energy analysis of solubilization in micelle. J Chem Phys 124:154908. 10.1063/1.2186324 16674266 10.1063/1.2186324

[39] Moffat AC, Osselton MD, Widdop B, Watts J (2011) Clarke’s analysis of drugs and poisons. Pharmaceutical press, London

[40] Florence AT, Attwood D, Florence AT, Attwood D (1998) The solubility of drugs. In: Florence AT, Attwood D (eds) Physicochemical principles of pharmacy. Macmillan Education UK, London, pp 152–198

[41] Glomme A, März J, Dressman JB (2006) Predicting the Intestinal Solubility of Poorly Soluble Drugs. In: Testa B, Krmer SD, Wunderli-Allenspach H, Folkers G (eds) Pharmacokinetic Profiling in Drug Research. Wiley, Weinheim, pp 259–280

[42] Zangenberg NH, Müllertz A, Gjelstrup Kristensen H, Hovgaard L (2001) A dynamic in vitro lipolysis model: II: evaluation of the model. Eur J Pharm Sci 14:237–244. 10.1016/S0928-0987(01)00182-8 11576829 10.1016/s0928-0987(01)00182-8

[43] Pluta RM, Jung CS, Harvey-White J et al (2005) In vitro and in vivo effects of probucol on hydrolysis of asymmetric dimethyl l-arginine and vasospasm in primates. J Neurosurg 103:731–738. 10.3171/jns.2005.103.4.0731 16266057 10.3171/jns.2005.103.4.0731

[44] Parrow A, Larsson P, Augustijns P, Bergström CAS (2020) Molecular dynamics simulations on interindividual variability of intestinal fluids: impact on drug solubilization. Mol Pharm 17:3837–3844. 10.1021/acs.molpharmaceut.0c00588 32787279 10.1021/acs.molpharmaceut.0c00588 PMC7704030

[45] van der Spoel D, Lindahl E, Hess B et al (2005) Gromacs user manual version 4.0. Nijenborgh 4:9747

[46] Pronk S, Páll S, Schulz R et al (2013) GROMACS 4.5: a high-throughput and highly parallel open source molecular simulation toolkit. Bioinformatics 29:845–854 23407358 10.1093/bioinformatics/btt055 PMC3605599

[47] Abraham MJ, Murtola T, Schulz R et al (2015) Gromacs: high performance molecular simulations through multi-level parallelism from laptops to supercomputers. SoftwareX 1–2:19–25. 10.1016/j.softx.2015.06.001

[48] Wang J, Wang W, Kollman PA, Case DA (2006) Automatic atom type and bond type perception in molecular mechanical calculations. J Mol Graph Model 25:247–260 16458552 10.1016/j.jmgm.2005.12.005

[49] Wang J, Wolf RM, Caldwell JW et al (2004) Development and testing of a general amber force field. J Comput Chem 25:1157–1174 15116359 10.1002/jcc.20035

[50] Jämbeck JPM, Lyubartsev AP (2012) Derivation and systematic validation of a refined all-atom force field for phosphatidylcholine lipids. J Phys Chem B 116:3164–3179. 10.1021/jp212503e 22352995 10.1021/jp212503e PMC3320744

[51] Jämbeck JPM, Lyubartsev AP (2012) An extension and further validation of an all-atomistic force field for biological membranes. J Chem Theory Comput 8:2938–2948. 10.1021/ct300342n 26592132 10.1021/ct300342n

[52] Jämbeck JPM, Lyubartsev AP (2013) Another piece of the membrane puzzle: extending slipids further. J Chem Theory Comput 9:774–784. 10.1021/ct300777p 26589070 10.1021/ct300777p

[53] Ermilova I, Lyubartsev AP (2016) Extension of the Slipids force field to polyunsaturated lipids. J Phys Chem B 120:12826–12842. 10.1021/acs.jpcb.6b05422 27966360 10.1021/acs.jpcb.6b05422

[54] Jorgensen WL, Chandrasekhar J, Madura JD et al (1983) Comparison of simple potential functions for simulating liquid water. J Chem Phys 79:926–935. 10.1063/1.445869

[55] Darden T, York D, Pedersen L (1993) Particle mesh Ewald: an N⋅ log (N) method for Ewald sums in large systems. J Chem Phys 98:10089–10092

[56] Essmann U, Perera L, Berkowitz ML et al (1995) A smooth particle mesh Ewald method. J Chem Phys 103:8577–8593

[57] Lundborg M, Lindahl E (2015) Automatic GROMACS topology generation and comparisons of force fields for solvation free energy calculations. J Phys Chem B 119:810–823 25343332 10.1021/jp505332p

[58] Vanquelef E, Simon S, Marquant G et al (2011) RED Server: a web service for deriving RESP and ESP charges and building force field libraries for new molecules and molecular fragments. Nucleic Acids Res 39:W511–W517 21609950 10.1093/nar/gkr288 PMC3125739

[59] Marrink SJ, Risselada HJ, Yefimov S et al (2007) The MARTINI force field: coarse grained model for biomolecular simulations. J Phys Chem B 111:7812–7824 17569554 10.1021/jp071097f

[60] de Jong DH, Singh G, Bennett WFD et al (2013) Improved parameters for the martini coarse-grained protein force field. J Chem Theory Comput 9:687–697 26589065 10.1021/ct300646g

[61] Hossain MS, Berg S, Bergström CAS, Larsson P (2019) Aggregation behavior of medium chain fatty acids studied by coarse-grained molecular dynamics simulation. AAPS PharmSciTech 20:61. 10.1208/s12249-018-1289-4 30627943 10.1208/s12249-018-1289-4 PMC6373435

[62] Martínez L, Andrade R, Birgin EG, Martínez JM (2009) PACKMOL: a package for building initial configurations for molecular dynamics simulations. J Comput Chem 30:2157–2164 19229944 10.1002/jcc.21224

[63] Bruce CD, Berkowitz ML, Perera L, Forbes MDE (2002) Molecular dynamics simulation of sodium dodecyl sulfate micelle in water: micellar structural characteristics and counterion distribution. J Phys Chem B 106:3788–3793

[64] Torrie GM, Valleau JP (1974) Monte Carlo free energy estimates using non-Boltzmann sampling: application to the sub-critical Lennard-Jones fluid. Chem Phys Lett 28:578–581

[65] Kumar S, Rosenberg JM, Bouzida D et al (1992) The weighted histogram analysis method for free-energy calculations on biomolecules. I. The method. J Comput Chem 13:1011–1021

[66] Hub JS, De Groot BL, Van Der Spoel D (2010) g\_wham A free weighted histogram analysis implementation including robust error and autocorrelation estimates. J Chem Theory Comput 6:3713–3720

[67] Khavrutskii IV, Dzubiella J, McCammon JA (2008) Computing accurate potentials of mean force in electrolyte solutions with the generalized gradient-augmented harmonic Fourier beads method. J Chem Phys 128:44106 10.1063/1.2825620 18247929

[68] Shirts MR, Chodera JD (2008) Statistically optimal analysis of samples from multiple equilibrium states. J Chem Phys 129:124105 19045004 10.1063/1.2978177 PMC2671659

[69] Elvang PA, Hinna AH, Brouwers J et al (2016) Bile salt micelles and phospholipid vesicles present in simulated and human intestinal fluids: structural analysis by flow field–flow fractionation/multiangle laser light scattering. J Pharm Sci 105:2832–2839 27103012 10.1016/j.xphs.2016.03.005

[70] Elvang PA, Jacobsen A-C, Bauer-Brandl A et al (2018) Co-existing colloidal phases in artificial intestinal fluids assessed by AF4/MALLS and DLS: a systematic study into cholate & (lyso-) phospholipid blends, incorporating celecoxib as a model drug. Eur J Pharm Sci 120:61–72 29704643 10.1016/j.ejps.2018.04.031

[71] Carey MC, Small DM (1972) Micelle formation by bile salts: physical-chemical and thermodynamic considerations. Arch Intern Med 130:506–527 4562149

[72] Mazer NA, Benedek GB, Carey MC (1980) Quasielastic light-scattering studies of aqueous biliary lipid systems. Mixed micelle formation in bile salt-lecithin solutions. Biochemistry 19:601–615 7356951 10.1021/bi00545a001

[73] Edueng K, Kabedev A, Ekdahl A et al (2021) Pharmaceutical profiling and molecular dynamics simulations reveal crystallization effects in amorphous formulations. Int J Pharm 613:121360. 10.1016/j.ijpharm.2021.121360 34896563 10.1016/j.ijpharm.2021.121360

[74] Kneiszl R, Hossain S, Larsson P (2022) In silico-based experiments on mechanistic interactions between several intestinal permeation enhancers with a lipid Bilayer model. Mol Pharm 19:124–137. 10.1021/acs.molpharmaceut.1c00689 34913341 10.1021/acs.molpharmaceut.1c00689 PMC8728740

[75] Souza PCT, Alessandri R, Barnoud J et al (2021) Martini 3: a general purpose force field for coarse-grained molecular dynamics. Nat Methods 18:382–388. 10.1038/s41592-021-01098-3 33782607 10.1038/s41592-021-01098-3 PMC12554258

[76] Miao M, Fu H, Zhang H et al (2021) Avoiding non-equilibrium effects in adaptive biasing force calculations. Mol Simul 47:390–394. 10.1080/08927022.2020.1775222

