# Supplementary material for: Molecular dynamics study on micelle-small molecule interactions: developing a strategy for an extensive comparison
Source: J Comput Aided Mol Des. 2023 Dec 16;38(1):5. doi: 10.1007/s10822-023-00541-1 (PMC10725378; doi:10.1007/s10822-023-00541-1)
Supplement: Supplementary file 1 — Supplementary file1 (DOCX 471 KB) [file 10822_2023_541_MOESM1_ESM.docx]

Journal of Computer-Aided Molecular Design

Appendix A for:

Molecular Dynamics Study on Micelle-Small Molecule Interactions: Developing a Strategy for an Extensive Comparison

Aleksei Kabedev ^1^, Christel A.S. Bergström^1,2^ and Per Larsson ^1,2,*^

^1^ Department of Pharmacy, Uppsala University, Uppsala, Sweden

^2^ Swedish Drug Delivery Center

***** Correspondence: per.r.larsson@uu.se

**Keywords:** molecular dynamics simulations, solubilization, extensive screening, umbrella sampling, intestinal fluid

**Fig. 1** Average and standard deviation evaluated with bootstrapping analysis for the self-assembled DLiPC (PL) micelles

**Fig. 2** Average and standard deviation evaluated with bootstrapping analysis for the pre-organised DLiPC (PL) micelles

**Fig. 3** Average and standard deviation evaluated with bootstrapping analysis for the self-assembled mixed sodium taurocholate-DLiPC (BS-PL) micelles

**Fig. 4** Average and standard deviation evaluated with bootstrapping analysis for the pre-organised mixed sodium taurocholate-DLiPC (BS-PL) micelles

**Fig. 5** Average and standard deviation evaluated with bootstrapping analysis for the self-assembled sodium taurocholate (BS) micelles

**Table 1** Summary of the simulation times required for different protocols. Here, equilibration time is excluded as it is normally the same between different setups and would be added to total times for each protocol. N_US-w_ and t_US-w_ are number and duration time of the umbrella sampling windows. N_lambda_ and t_lambda_ are number and duration time of the lambda states for point-wise free energy calculations. 20 cpu and 480 cpu are taken as reference values, as these correspond approximately to the amount of resources used in parallel to perform the simulations.

|  | Computational protocol | | | | | | |
| --- | --- | --- | --- | --- | --- | --- | --- |
|  | AA | | | | | CG | FEP |
|  | Self-assembled colloids | | | Pre-organised colloids | | Self-assembled colloids | Pre-organised colloids |
|  | BS | PL | Mixed | PL | Mixed | Mixed | PL |
| Calculation scheme | N_US-w_ * t_US-w_ | | | | | CG mapping + N_US-w_ * t_US-w_ | N_FEP_ * N_lambda_ * t_Lambda_ |
| Performance,  ns/day,  estimate | 24 | 21 | 20 | 16.5 | 5.8 | 8,200 | 11.4 |
| N_US-w_ \| N_lambda_ | 50 | 35 | 40 | 55 | 70 | 55 | 20 |
| t_US-w_, ns | 20 | 20 | 20 | 20 | 20 | 900 | 20 |
| Real time per simulation, hours, on 20 cpu, without equilibration | 20 | 22.9 | 24 | 29.1 | 82.8 | 2.9 | 1.8 |
| Total series time, 480 cpu, without equilibration | 60 | 45.7 | 48 | 87.3 | 248.3 | 8.8 | 5.3 |
